# Resistant hypertension and cardiovascular disease mortality in the US: results from the National Health and Nutrition Examination Survey (NHANES)

**DOI:** 10.1186/s12882-019-1315-0

**Published:** 2019-04-25

**Authors:** Katerina R. Kaczmarski, Stephen M. Sozio, Jingsha Chen, Yingying Sang, Tariq Shafi

**Affiliations:** 1 Department of Medicine, Johns Hopkins University School of Medicine, 600 N. Wolfe St, Baltimore, MD 21287 USA; 2 Welch Center for Prevention, Epidemiology and Clinical Research, Johns Hopkins Medical Institutions, Baltimore, MD USA; 3 Department of Biostatistics, Johns Hopkins Bloomberg School of Public Health, Baltimore, MD USA; 4 Department of Medicine, University of Mississippi Medical Center, Jackson, MS USA

**Keywords:** Hypertension, Anti-hypertensives, Cardiovascular mortality, All-cause mortality

## Abstract

**Background:**

Apparent treatment-resistant hypertension (aTRH) is a common condition associated with risk of cardiovascular events. However, the risk of cardiovascular mortality associated with aTRH in the US population is unknown. We aimed to assess the risk of cardiovascular disease (CVD) mortality associated with aTRH in the US population.

**Methods:**

We analyzed data from 6357 adult hypertensive participants of the National Health and Nutrition Examination Survey (1988–1994 and 1999–2010) linked to the National Death Index. Based on presence of uncontrolled hypertension [blood pressure (BP) ≥140/90 mmHg] and the number of antihypertensives prescribed, we classified participants into the following groups: non-aTRH (BP < 140/90 mmHg and ≤ 3 antihypertensives); controlled aTRH (BP < 140/90 mmHg and ≥ 4 antihypertensives); and uncontrolled aTRH (BP ≥140/90 mmHg and ≥ 3 antihypertensives).

**Results:**

Of the 6357 participants, 1522 had aTRH, representing a US prevalence of 7.6 million. Of the participants with aTRH, 432 had controlled aTRH and 1090 had uncontrolled aTRH. During follow-up (median 6 years), there were 550 CVD deaths. The cumulative incidence of CVD mortality was significantly higher in the aTRH group compared with non-aTRH group (log-rank *p* < 0.001). In fully adjusted models, aTRH was associated with a 47% higher risk of CVD mortality compared with the non-aTRH group [1.47 (1.1–1.96)]. Similar increase in risk of CVD mortality was noted across aTRH subgroups compared with the non-aTRH group: controlled aTRH [1.66 (1.03–2.68)] and uncontrolled aTRH [1.43 (1.05–1.94)]. Among non-aTRH subgroups, those on 3 antihypertensive medications had a 35% increased risk of CVD mortality than those on < 3 medications [1.35 (0.98–1.86)].

**Conclusions:**

aTRH is a common condition, affecting approximately 7.6 million Americans. Regardless of BP control, people with aTRH remain at a higher risk of cardiovascular outcomes. The risk of cardiovascular disease mortality remains high among those with controlled BP on 3 medications (non-aTRH) or ≥ 4 medications (controlled aTRH), groups not generally considered at high risk. Future risk reduction interventions should consider focusing on these high-risk groups.

**Electronic supplementary material:**

The online version of this article (10.1186/s12882-019-1315-0) contains supplementary material, which is available to authorized users.

## Key points

**Question**: What is the risk of cardiovascular disease (CVD) mortality associated with apparent treatment resistant hypertension (aTRH) in the US population?

**Findings**: In this retrospective case-control study that included 6357 adults, aTRH was associated with a 47% higher risk of cardiovascular mortality compared with the non-aTRH group. aTRH has a US prevalence of 7.6 million people.

**Meaning**: Regardless of BP control, people with aTRH remain at a higher risk of cardiovascular outcomes; future risk reduction interventions should consider focusing on these high-risk groups.

## Background

Hypertension affects 29% of the US adult population, corresponding to approximately 90 million Americans [1]. Hypertension is a risk factor for a number of adverse cardiovascular outcomes, including stroke, coronary heart disease, and heart failure [2]. Treatment of hypertension prevents and reduces cardiovascular morbidity, notably a 40% reduction in risk of stroke and 15% reduction in risk of myocardial infarction [3, 4]. Recent data suggest that hypertension awareness has improved with a reduction in the proportion of untreated hypertensive patients; however, the percentage of patients on multiple antihypertensive medications has progressively risen [5].

Apparent treatment-resistant hypertension (aTRH) is defined as hypertension that remains uncontrolled (≥140/≥90 mmHg) despite treatment with ≥3 antihypertensive medications or hypertension that is controlled (< 140/90 mmHg) on ≥4 antihypertensives [6]. In comparison with non-aTRH hypertensive patients, patients with aTRH, regardless of blood pressure (BP) control, have a higher risk of coronary artery disease, cardiovascular disease, heart failure, end-stage renal disease (ESRD), and all-cause mortality [7–10]. With the rise in prevalence of hypertensive patients with aTRH from 5.5% in 1988 to 11.8% in 2008, aTRH has become a common condition within the US population [5]. However, the risk of cardiovascular mortality associated with aTRH in the US population is unknown.

The goal of our study was to assess the risk of cardiovascular mortality amongst individuals with aTRH in the US using data from the National Health and Nutrition Examination Survey. Furthermore, we sought to compare the risk of cardiovascular mortality amongst aTRH subgroups, stratified by BP control.

## Methods

### Study sample

**Fig. 1:**
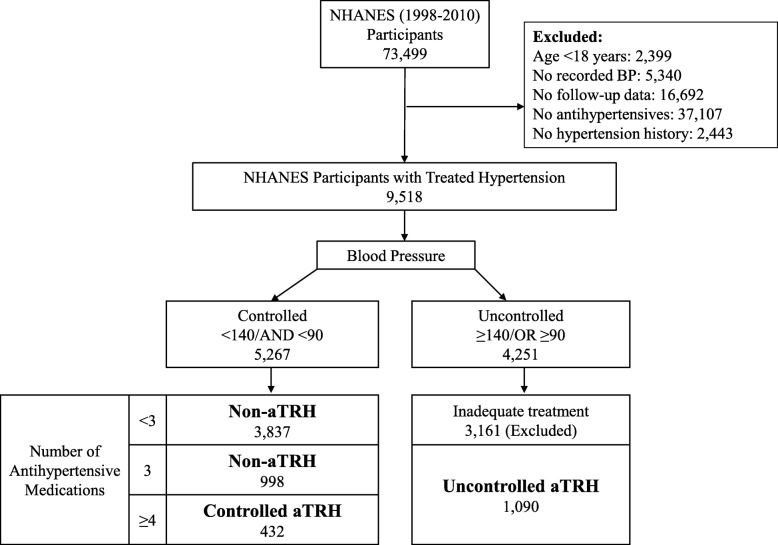
Selection of the study population

The NHANES are cross-sectional, multistage, stratified, clustered probability sample of the noninstitutionalized US civilian population conducted by the National Center for Health Statistics (NCHS), a branch of the Center for Disease Control and Prevention. NHANES III was conducted from 1988 to 1994 and the continuous NHANES were conducted from 1999 to 2010 with data released in 2 year cycles. We excluded participants that were < 18 years of age, had no recorded BP measurements, no follow-up, no antihypertensive use, and no known diagnosis of hypertension (Fig. 1). We further excluded persons with inadequately treated uncontrolled hypertension defined as BP ≥140/90 mmHg and use of < 3 antihypertensive medications because these patients are not considered to have aTRH based on current definitions of treatment resistant hypertension, but we cannot exclude the presence of aTRH given inadequate antihypertensive treatment. After these exclusions, 6357 participants were included in the final study population. The protocols for conduct of NHANES were approved by the NCHS institutional review board and informed consent was obtained from all participants. The Johns Hopkins Medical Institutions Institutional Review Board reviewed and approved the study.

### BP, Antihypertensives and definition of treatment resistant hypertension

BP was measured after the participant had been resting in a seated position for 5 min with feet on the floor. Three consecutive BP readings were obtained and reported as the mean of all systolic readings over the mean of all diastolic readings [11]. Antihypertensive medication use was self-reported, and antihypertensive medications were classified as diuretics, angiotensin converting enzyme inhibitors, angiotensin receptor blockers, beta blockers, calcium channel blockers, and others according to classification codes provided with the NHANES data. Medication dose data was not available. We defined uncontrolled hypertension as systolic BP ≥140 mmHg and/or diastolic BP ≥90 mmHg. Using BP control and number of antihypertensive medications, we classified participants into the following groups:

- Non-aTRH: Hypertensive participants with controlled hypertension (BP < 140/< 90 mmHg) treated with ≤3 medications. We further subdivided this group into those on 3 antihypertensives and those on < 3 antihypertensives.
- Controlled aTRH: Hypertensive participants with controlled hypertension (BP < 140/< 90 mmHg) treated with ≥4 antihypertensives.
- Uncontrolled aTRH: Hypertensive participants with uncontrolled hypertension (BP ≥140/90 mmHg) treated with ≥3 antihypertensives.

### Measurements – other variables

Standardized questionnaires were administered at home, and physical examination and laboratory test specimen collection were performed at the mobile examination center. Self-reported race/ethnicity was categorized as non-Hispanic White, non-Hispanic Black, Mexican-American or other. Smoking was defined as either ever smoked or never having smoked. Participants were considered to have diabetes mellitus if they reported being told by a doctor that they had diabetes at a time other than pregnancy or if they were taking insulin or oral hypoglycemic agents. Cardiovascular disease was considered to be present at baseline if the participant reported being informed by a doctor of prior heart attack, congestive heart failure or stroke. Body mass index was calculated by dividing weight in kg by height in m^2^ and modeled as a linear spline with knots at 18 kg/m^2^ and 25 kg/m^2^. Serum creatinine was measured using a kinetic rate Jaffe method and used to calculate estimated glomerular filtration rate (eGFR) [12], and modeled as a linear spline with knots at 60 and 90 ml/min/1.73 m^2^. Total cholesterol levels included both fasting and non-fasting samples and were modeled as a continuous variable. C-reactive protein was measured by latex-enhanced nephelometry (Dade Behring). Urinary albumin was measured by solid-phase fluorescence immunoassay, and urinary creatinine was measured by the modified kinetic method of Jaffe using a Beckman Coulter Synchron AS/Astra Analyzer (Beckman Coulter, Inc., Fullerton, California). C-reactive protein and urinary albumin-to-creatinine ratio were modeled as continuous variables after natural log transformation.

### Causes of death

Causes of death were obtained using the NHANES Public-use Linked Mortality Files [11]. This file contains mortality follow-up data on NHANES participants obtained via National Death Index (NDI) linkage through December 31, 2011. The underlying cause of death was coded using the UCOD_LEADING variable and was classified as either all-cause death or death due to cardiovascular disease (UCOD_LEADING values 001 or 005) [11].

### Statistical analysis

All analyses were performed incorporating sampling that account for the complex survey design of NHANES, allowing generation of nationally representative estimates for the US population. Population estimates were standardized to the 2017 U.S. population. Baseline characteristics were compared among those with controlled hypertension versus aTRH and among subgroups of aTRH. Survival analysis techniques were used to analyze the risk of mortality associated with aTRH. Individuals who were alive on December 31, 2011 were censored in the analyses. Kaplan-Meier survival plots were used to assess the differences in survival among those with controlled hypertension and aTRH categories. Poisson regression was used to calculate mortality rates. Modified Cox proportional hazards regression was used to model the risk of death with non-aTRH participants as the reference group. Hazard ratios (HR) were calculated for unadjusted models (Model 1) and after sequentially adjusting for age, race/ethnicity, sex (Model 2), followed by further adjustment for diabetes and cardiovascular disease, smoking status, body mass index, serum total cholesterol, and C-reactive protein (CRP) (Model 3). The fully adjusted model (Model 4) further adjusted for estimate glomerular filtration rate (eGFR) and albumin-to-creatinine ratio (ACR). Pre-specified subgroup analyses included categories of age, sex, race/ethnicity, smoking status, diabetes, cardiovascular disease, diuretic use, eGFR, CRP and ACR. All analyses were performed using survey (svy) commands in Stata 13.1 (Stata Corp, www.stata.com). A *p*-value of < 0.05 was considered statistically significant.

## Results

### Baseline participant characteristics

Figure 1 describes the selection of the final study population. Of the 6357 NHANES participants included in this analysis, 1522 had aTRH. Within the US population, this corresponds to a prevalence of 7.6 million adults with aTRH, of which 2.27 million have controlled aTRH, and 5.33 million have uncontrolled aTRH (Table 1). The prevalence of hypertensive US adults without aTRH is 29.04 million, of which 23.49 million have controlled BP on < 3 medications, and 5.55 million have controlled BP on 3 medications. Note that these prevalence estimates exclude people with inadequately treated (uncontrolled on < 3 medications) or untreated hypertension. The baseline characteristics of the hypertensive NHANES participants with and without aTRH are described in Table 1. Compared to the participants without aTRH, those with aTRH were older (66 years versus 59 years) and more likely to be African-American, have a history of diabetes and prior cardiovascular disease, and have a lower eGFR. Among aTRH versus non-aTRH participants, 86.8% vs. 46.3% were on a diuretic, 83.2% vs. 63.4% were on an ACE-I or ARB, 56.6% vs. 24.5% were on a calcium channel blocker, and 66.6% vs. 34.6% were on a beta blocker.

**Table 1:** Baseline Characteristics of Included NHANES Participants

Characteristic^a^	Non-aTRH			aTRH		
	Any Non-aTRH	Non-aTRH Subgroups		Any aTRH	aTRH Subgroups	
		< 3 medications	3 medications		Controlled	Uncontrolled
Definition: BP mm Hg and number of antihypertensives	< 140/90 AND ≤3	< 140/90 AND < 3	< 140/90 AND 3	> 140/90 OR ≥3	< 140/90 AND ≥4	≥140/90 AND ≥3
Unweighted Population (N)	4835	3837	998	1522	432	1090
US Weighted Population (N in Millions)^b^	29.04	23.49	5.55	7.6	2.27	5.33
Age (years), mean (SE)	59.4 (0.3)	58.3 (0.3)	64 (0.3)	65.9 (0.5)	65.1 (0.6)	66.2 (0.8)
Age Categories						
Age < 45 years	510 (14.1)	457 (15.5)	53 (8)	69 (5.9)	14 (4.6)	55 (6.5)
Age 45–65 years	2055 (48.8)	1709 (51)	346 (39.3)	456 (35.5)	138 (38.7)	318 (34.2)
Age ≥ 65 years	2270 (37.1)	1671 (33.4)	599 (52.8)	997 (58.5)	280 (56.7)	717 (59.3)
Sex						
Female	2607 (55.1)	2094 (55.3)	513 (54.2)	819 (57.4)	209 (52.5)	610 (59.5)
Male	2228 (44.9)	1743 (44.7)	485 (45.8)	703 (42.6)	223 (47.5)	480 (40.5)
Race/Ethnicity						
Non-Hispanic White	2645 (78.3)	2073 (78.5)	572 (77.3)	787 (73.4)	224 (74.4)	563 (73)
Non-Hispanic Black	1221 (12.3)	959 (12)	262 (13.8)	491 (18.3)	147 (18.1)	344 (18.4)
Mexican American	823 (5.4)	685 (5.4)	138 (5.7)	210 (5)	51 (4.7)	159 (5.1)
Other	146 (4)	120 (4.2)	26 (3.3)	34 (3.3)	10 (2.8)	24 (3.5)
Ever or Current Smoker	2643 (55.1)	2077 (55.3)	566 (53.8)	838 (53.1)	257 (58.4)	581 (50.8)
Diabetes	1459 (24.5)	1090 (22.7)	369 (32.4)	661 (39.5)	210 (44.1)	451 (37.5)
Prior CVD	1101 (18.9)	750 (15.9)	351 (31.4)	636 (40.1)	213 (48.5)	423 (36.4)
Body mass index (kg/m^2^), mean (SE)	30.8 (0.1)	30.6 (0.1)	31.8 (0.3)	32.1 (0.3)	33.2 (0.5)	31.7 (0.3)
Systolic blood pressure (mmHg), mean (SE)	122.2 (0.2)	122.3 (0.2)	121.7 (0.4)	144.7 (0.7)	119.7 (0.7)	155.4 (0.6)
Diastolic blood pressure (mmHg), mean (SE)	69.6 (0.3)	70.3 (0.3)	66.6 (0.5)	71.1 (0.5)	63.6 (0.9)	74.3 (0.6)
# of anti-hypertensives, mean (SE)	1.77 (0.01)	1.48 (0.01)	3 (0)	3.67 (0.03)	4.26 (0.03)	3.41 (0.03)
Diuretics	2333 (46.3)	1512 (37.5)	821 (83.5)	1310 (86.8)	404 (93.5)	906 (83.9)
ACE-i or ARB	2563 (63.4)	1862 (59.8)	701 (77.9)	1115 (83.2)	370 (89.3)	745 (80.2)
Calcium channel blockers	1348 (24.5)	890 (20.2)	458 (43)	892 (56.6)	283 (65.1)	609 (53)
Beta blockers	1555 (34.6)	997 (29)	558 (58.1)	991 (66.6)	326 (77.2)	665 (62)
Other	481 (8)	281 (5.7)	200 (17.5)	624 (38.1)	211 (45.9)	413 (34.7)
Total Cholesterol (mmol/L), mean (SE)	5.2 (0.02)	5.2 (0.03)	5.1 (0.05)	5.2 (0.05)	4.8 (0.08)	5.3 (0.05)
eGFR (mL/min/1.73 m^2^), median (IQR)	82 (66, 96)	84 (69, 97)	71 (58, 87)	68 (51, 85)	67 (52, 86)	68 (51, 85)
CRP (mg/dL), mean (SE)	0.56 (0.02)	0.54 (0.02)	0.65 (0.04)	0.68 (0.03)	0.67 (0.05)	0.68 (0.04)
ACR (mg/g), median (IQR)	7 (5, 15)	7 (5, 13)	9 (5, 22)	14 (6, 49)	10 (6, 26)	17 (7, 67)

Data are presented as N (%) and mean (SE), unless otherwise indicated

*Abbreviations*: *USRDS* United States Renal Data System, *SE* Standard Error, *CVD* Cardiovascular Disease, *eGFR* estimated Glomerular Filtration Rate, *IQR* Interquartile Range, *CRP* C-reactive Protein, *ACR* Albumin-Creatinine Ratio, *aTRH* apparent treatment resistant hypertension, *ACE-i* angiotensin converting enzyme inhibitors, *ARB* angiotensin receptor blockers, *SE* standard error

^a^ Unweighted refers to the actual number (%) of the NHANES participants from the final cohort

^b^ Weighted refers to the projected numbers in the US populations and accounts for NHANES design and sampling weights

Baseline characteristics were similar between aTRH subgroups. Notably, the controlled aTRH subgroup had a greater percentage of participants with prior CVD (48.5% vs. 36.4%), defined as prior heart attack, congestive heart failure, or stroke, compared to the uncontrolled aTRH subgroup. Compared to non-aTRH patients on < 3 medications, patients on 3 medications were more likely to have diabetes (32.4% vs. 22.7%), prior CVD (31.4% vs. 15.9%), and a prescription for a diuretic (83.5% vs. 37.5%).

### aTRH and risk of cardiovascular mortality

There were 541 deaths due to cardiovascular disease during follow-up (median 6 years). The crude cardiovascular mortality rate (Table 2) was over 2-fold higher in the aTRH group (19.4 per 1000 person-years) compared with the non-aTRH group (7.4 deaths per 1000 person-years). Within the aTRH subgroups, the crude cardiovascular mortality was similar between the controlled aTRH subgroup (19.1 per 1000 person-years) and uncontrolled aTRH subgroup (19.5 per 1000 person-years). Within the non-aTRH subgroups, the crude cardiovascular mortality was 2-fold greater in the participants on 3 medications (13 per 1000 person-years) in comparison to those on < 3 medications (6.3 per 1000 person-years).

**Table 2:** Association of aTRH with CVD Mortality among NHANES (1988–1994 and 1999–2010) Participants

	Unweighted^a^		Weighted^b^		Crude IR^b^ (95% CI) Per 1000 PY	Model 1: Unadjusted^c^		Model 2^d^		Model 3^e^		Model 4: Fully Adjusted^f^	
	N (%)	Deaths (%)	N in millions (%)	Deaths in millions (%)		HR	p	HR	p	HR	p	HR	p
Non-aTRH	4835 (76.1)	339 (7)	29.04 (79.3)	1.6 (5.5)	7.4 (6.4, 8.5)	Reference		Reference		Reference		Reference	
Any aTRH	1522 (23.9)	211 (13.9)	7.6 (20.7)	0.97 (12.8)	19.4 (16.2, 23.4)	2.69 (2.14, 3.4)	< 0.001	1.85 (1.47, 2.34)	< 0.001	1.73 (1.34, 2.23)	< 0.001	1.47 (1.1, 1.96)	0.01
Controlled	432 (6.8)	45 (10.4)	2.27 (6.2)	0.22 (9.9)	19.1 (13.1, 27.8)	2.82 (1.91, 4.15)	< 0.001	2.06 (1.41, 3.03)	< 0.001	1.78 (1.12, 2.81)	0.015	1.66 (1.03, 2.68)	0.039
Uncontrolled	1090 (17.1)	166 (15.2)	5.33 (14.5)	0.75 (14.1)	19.5 (16, 23.9)	2.66 (2.07, 3.41)	< 0.001	1.8 (1.41, 2.29)	< 0.001	1.72 (1.32, 2.24)	< 0.001	1.43 (1.05, 1.94)	0.025
						p-trend	< 0.001	p-trend	< 0.001	p-trend	< 0.001	p-trend	0.017

*Abbreviations*: *aTRH* apparent treatment-resistant hypertension, *IR* incidence ratio

^a^Unweighted refers to the actual number (%) of the NHANES participants from the final cohort

^b^Weighted refers to the projected numbers in the US populations and accounts for NHANES design and sampling weights

HR from Cox proportional hazards models with adjustment as follows:

^c^Model 1 was unadjusted

^d^Model 2 adjusted for age, sex and race

^e^Model 3 adjusted for variables in Model 2 + body mass index (spline at 18 kg/m^2^ and 25 kg/m^2^), history of diabetes and cardiovascular disease, smoking status, serum total cholesterol, and ln-C-reactive protein (natural log transformed)

^f^Model 4 adjusted for variables in Model 3 + eGFR CKD EPI (spline at 60 and 90) and ln-ACR

**Fig. 2:**
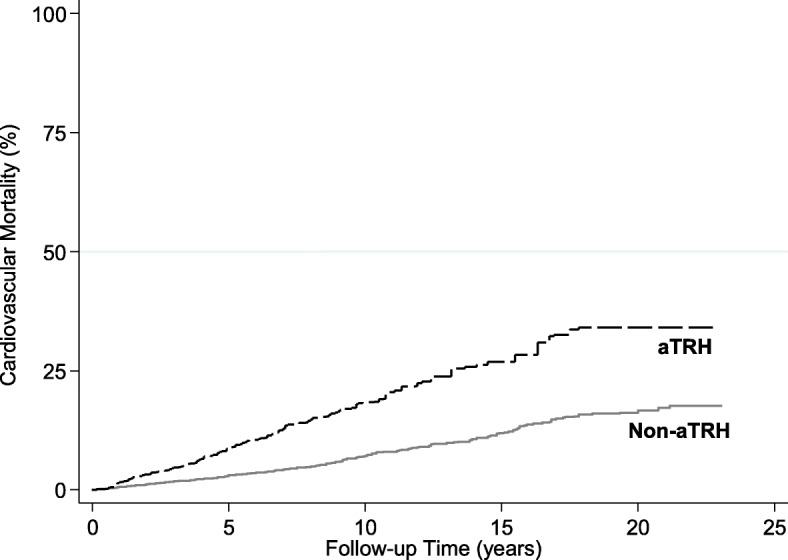
Cumulative Incidence of Cardiovascular Disease Mortality among NHANES participants with aTRH and controlled hypertension

The cumulative cardiovascular mortality in the non-aTRH and aTRH groups is displayed in Fig. 2. Those with aTRH, both controlled and uncontrolled, had a significantly higher cardiovascular mortality than those in the non-aTRH groups (log-rank *p* < 0.001).

The unadjusted and adjusted models for risk of cardiovascular mortality are presented in Table 2. In Model 3, adjusting for age, race/ethnicity, sex, body mass index, history of diabetes and cardiovascular disease, smoking status, and serum total cholesterol and CRP, people with aTRH had a 73% higher risk of cardiovascular mortality compared with the non-aTRH group [1.73 (1.34–2.23)]. After further adjustment (Model 4), accounting for eGFR and ACR (Table 2), people with aTRH had a 47% higher risk of cardiovascular mortality compared with the non-aTRH group [1.47 (1.1–1.96)]. Similarly, aTRH subgroups had a higher risk of cardiovascular mortality in comparison to the non-aTRH group: controlled aTRH [1.66 (1.03–2.68)] and uncontrolled aTRH [1.43 (1.05–1.94)]. Among non-aTRH subjects, those on 3 antihypertensives had a trend toward greater risk of cardiovascular mortality than those on < 3 antihypertensives [1.35 (0.98–1.86)] (Table 3).

**Table 3:** CVD Mortality among Non-aTRH NHANES (1988–1994 and 1999–2010) Participants

	Unweighted^a^		Weighted^b^		Crude IR (Weighted)	Model 1: Unadjusted^c^		Model 2^d^		Model 3^e^		Model 4: Fully Adjusted^f^	
	N (%)	Deaths (%)	N in millions (%)	Deaths in millions (%)	Per 1000 PY	HR	p	HR	p	HR	p	HR	p
Non-aTRH < 3 medications	3837 (60.4)	243 (6.3)	23.49 (64.1)	1.14 (4.9)	6.3 (5.4, 7.5)	Reference		Reference		Reference		Reference	
Non-aTRH 3 medications	998 (15.7)	96 (9.6)	5.55 (15.1)	0.46 (8.3)	13 (9.9, 16.9)	2.14 (1.55, 2.95)	< 0.001	1.59 (1.18, 2.16)	0.003	1.57 (1.15, 2.15)	0.005	1.35 (0.98, 1.86)	0.064

*Abbreviations*: *aTRH* apparent treatment-resistant hypertension, *IR* incidence ratio

^a^ Unweighted refers to the actual number (%) of the NHANES participants from the final cohort

^b^ Weighted refers to the projected numbers in the US populations and accounts for NHANES design and sampling weights

HR from Cox proportional hazards models with adjustment as follows:

^c^Model 1 was unadjusted

^d^Model 2 adjusted for age, sex and race

^e^Model 3 adjusted for variables in Model 2 + body mass index (spline at 18 kg/m^2^ and 25 kg/m^2^), history of diabetes and cardiovascular disease, smoking status, serum total cholesterol, and ln-C-reactive protein (natural log transformed)

^f^Model 4 adjusted for variables in Model 3 + eGFR CKD EPI (spline at 60 and 90) and ln-ACR

### All-cause mortality

In Model 3, people with aTRH had a 33% increased risk of all-cause mortality compared with the non-aTRH group [1.33 (1.13–1.57)]. In the fully adjusted model (Model 4), there was no significant increased risk of all-cause mortality of the aTRH group in comparison to the non-aTRH group [1.15 (0.97–1.36)] (Additional file 1: Table S1).

### Subgroup analyses

Pre-specified subgroup analyses are presented in Table 4. Due to multiple comparisons, a p-interaction of < 0.005 (*p* = 0.05/10) is suggested as a significant value. Using this threshold, there were no significant differences within subgroups.

**Table 4:** Subgroup Analysis for Cardiovascular Mortality Associated with aTRH

	N	Deaths	Any aTRH vs. Controlled	p-interaction
Age (years)				
< 45 years	579	15	NA	0.211
45–65 years	2511	117	2.13 (1.18, 3.88)	
≥ 65 years	3267	418	1.23 (0.91, 1.66)	
Sex				
Female	3426	269	1.87 (1.26, 2.79)	0.033
Male	2931	281	1.13 (0.76, 1.69)	
Race/Ethnicity				
Non-Hispanic White	3432	343	1.56 (1.13, 2.15)	
Non-Hispanic Black	1712	143	1.17 (0.73, 1.88)	0.583
Mexican American	1033	58	1.15 (0.38, 3.43)	
Other	180	6	NA	
Nonsmoker	2869	218	2.16 (1.34, 3.47)	0.075
Smoker	3481	332	1.19 (0.85, 1.65)	
No Diabetes	4237	349	1.48 (1.05, 2.07)	0.746
Diabetes	2120	201	1.46 (0.94, 2.28)	
No CVD	4558	261	1.68 (1.1, 2.56)	0.03
CVD	1737	282	1.35 (1, 1.81)	
No Diuretic	2714	161	1.223 (0.62, 2.4)	0.818
Diuretic	3643	389	1.48 (1.04, 2.11)	
No ACE-I or ARB	1705	101	1.35 (0.69, 2.62)	0.562
ACE-I or ARB	3678	260	1.7 (1.18, 2.44)	
No CCB^b^	4117	303	1.54 (0.98, 2.43)	0.549
CCB^b^	2240	247	1.35 (0.93, 1.98)	
No Betablocker	3811	309	1.51 (1.03, 2.22)	0.411
Betablocker	2546	241	1.4 (0.91, 2.16)	
No Antihtn_other^c^	5252	411	1.41 (0.98, 2.01)	0.649
Antihtn_other^c^	1105	139	1.55 (0.89, 2.69)	
eGFR CKDEPI Categories^a^				
≥ 120	135	5	NA	0.521
90–119	1617	47	1.6 (0.57, 4.47)	
60–89	2677	215	1.34 (0.88, 2.04)	
45–59	929	129	1.94 (1.17, 3.2)	
30–44	417	76	1.08 (0.47, 2.47)	
< 30	187	38	2.33 (0.32, 16.86)	
CRP (mg/dL)				
< 0.22	2546	220	1.18 (0.76, 1.84)	0.582
0.22–0.99	2487	187	1.74 (1.16, 2.6)	
≥ 1.00	981	109	2.17 (0.99, 4.77)	
ACR				
< 30	4807	310	1.79 (1.27, 2.52)	0.063
30–300	1061	142	1.31 (0.83, 2.07)	
≥ 300	292	57	0.93 (0.43, 2)	

*Abbreviations*: *aTRH* apparent treatment-resistant hypertension, *CVD* Cardiovascular Disease, *eGFR CKD-EPI* estimated Glomerular Filtration Rate using Chronic Kidney Disease Epidemiology Collaboration, *CRP* C-reactive Protein, *ACR* Albumin-Creatinine Ratio

^a^ measured in mL/min/1.73 m^2^

^b^ Calcium channel blocker

^c^ Other anti-hypertensive not included in the preceding anti-hypertensive classes

## Discussion

In this U.S. nationally-representative study, we found that 7.6 million US adults with diagnosed and treated hypertension had aTRH, which was associated with 47% higher risk of cardiovascular mortality compared to people without aTRH. Those with controlled aTRH and uncontrolled aTRH had 66 and 43% higher risk of cardiovascular death, compared with the non-aTRH group. Among participants without aTRH, those on 3 antihypertensive medications had a trend toward higher risk of cardiovascular mortality than those on < 3 antihypertensive medications. Our findings suggest that patients with aTRH and non-aTRH requiring 3 medications for BP control represent high cardiovascular disease risk groups and should be considered as targets for risk reduction interventions.

Hypertension increases the risk of coronary artery disease, myocardial infarction, stroke, accelerated atherosclerosis, congestive heart failure, and ESRD [13]. While microvascular changes play a role in the pathogenesis of hypertension, uncontrolled hypertension is known to cause further microvascular structural and functional alterations, notably smooth muscle cell hypertrophy and collagen deposition in resistance arterioles [14]. Hypertension is also associated with conditions, such as obesity and diabetes mellitus, that further alter and damage microvasculature in presence of an inflammatory milieu [15], resulting in impaired endothelial insulin signaling and insulin-stimulated nitric oxide synthesis [16]. Similarly, the aTRH participants in our study were more likely to have diabetes (39.5% vs. 24.5%) and CVD (40.1% vs. 18.9%) than the non-aTRH participants. These comorbidities may have influenced the hypertensive disease burden and blood pressure control in aTRH participants. It is important to consider the differences in the baseline characteristics between the aTRH and non-aTRH groups when interpreting our results. However, the baseline characteristics seen in our study are representative of that of the US population, thus the findings of our study are applicable to general practice.

Over the past three decades, significant efforts have been focused on improving awareness, treatment, and control of hypertension. According to Egan et al., the percentage of hypertensive patients with uncontrolled hypertension decreased from 73.2% in 1988 to 52.5% in 2008 [5]. However, with the reduction in uncontrolled hypertension and increased awareness of treatment, there has been an increase in the prevalence of aTRH. Between 1988 and 2008, the percentage of hypertensive patients on 1 or 2 medications remained relatively constant, while the percentage of patients on ≥3 medications increased progressively [5]. It is generally assumed that once the BP is at goal, regardless of the number of medications being used, the risk of cardiovascular disease is mitigated. However, our study demonstrates that despite adequate blood pressure control, people with aTRH are at an increased risk of cardiovascular mortality.

In prior studies, aTRH was found to be associated with higher risk of ESRD, ischemic cardiac events, congestive heart failure, and stroke in comparison to patients with non-resistant hypertension [7, 17]. Importantly, cardiovascular disease risk remains elevated amongst patients with aTRH, regardless of BP control [7, 17]. In a study by Muntner et al., 14,684 ALLHAT trial participants were categorized as having aTRH or non-resistant hypertension. There was a higher risk of coronary heart disease, stroke, all-cause mortality, heart failure, and ESRD in those with aTRH [7]. Similarly, in a study by Irvin et al., a higher risk of coronary heart disease, stroke, and all-cause mortality was noted among those with aTRH [18]. Our study similarly demonstrates increased risk of CVD mortality amongst people with aTRH, therefore extending the findings from prior studies with data generalizable to the US population. However, unlike prior studies [8–10, 18], our study did not show a significantly increased risk of all-cause mortality in the aTRH group in comparison to the non-aTRH group. This suggests that individuals with aTRH are at greater risk of cardiovascular-related mortality but overall have similar risk of all-cause mortality.

Although the pathophysiology of aTRH is not well-understood, it is known that patients with aTRH have higher levels of brain-type and natriuretic peptides as well as aldosterone [19]. Additional contributing factors to aTRH may include non-adherence with lifestyle changes, including diet and exercise, and inadequate pharmacologic therapy [20]. Pharmacologic management of apparent treatment-resistant hypertension should include therapeutic optimization of antihypertensive dosages, systematic measurement of BP response to treatment, and assistance in overcoming barriers to adherence. Our study shows that even with medication prescription and BP control, aTRH patients remain at a higher risk of cardiovascular outcomes, suggesting a need for further risk mitigation strategies. The ongoing TRIUMPH trial (Clinicaltrials.gov no. NCT02342808) is evaluating the efficacy of lifestyle intervention using sodium restriction, DASH diet, exercise, weight management, and patient instruction and education in the treatment of aTRH [21]. Experimental treatments include renal denervation and electrical carotid baroreceptor stimulation using implantable devices; however, further studies are necessary for determination of efficacy and safety for use in treatment of aTRH [22, 23].

The observed associations between aTRH and cardiovascular mortality in our study provides some interesting insights (Table 2). In the unadjusted model (Model 1), aTRH was associated with a 2.5-fold higher risk of cardiovascular mortality, compared to the non-aTRH group. Adjusting for age, sex, and race (Model 2) reduces the magnitude of this association, reflecting the confounding effects of these variables. The association is further attenuated by adjusting for additional confounders in Model 3, in particular baseline diabetes and cardiovascular disease which had higher prevalence in the aTRH group as compared to the non-aTRH group. In Model 4, adjusting for kidney function (estimated glomerular filtration rate and albuminuria), further attenuates the association. It is likely that some of these adjustment factors, such as baseline cardiovascular disease and kidney function mediate the observed association between aTRH and outcomes. The higher systolic BP in the aTRH group (144 mmHg) as compared to the non-aTRH group (122 mmHg) could be contributing to higher prevalence of cardiovascular and kidney disease noted at the baseline in the aTRH group (Table 1). At baseline 40.1% of those in the aTRH group had cardiovascular disease, compared to 18.9% in the non-aTRH group. Similarly, the aTRH group had lower estimated glomerular filtration rate (68 ml/min|1.73 m^2^) as compared to the non-aTRH group (82 ml/min|1.73 m^2^). Clinical implication of these findings is that in patients with aTRH (whether controlled or uncontrolled) careful attention must be paid to reducing cardiovascular risk and preserving kidney function. From a population health perspective, aTRH identifies a patient population at 2.5-fold higher risk of cardiovascular mortality. This high-risk population can be easily identified using electronic health records, and population health management strategies could target this population for focused interventions such as lifestyle modification, and use of medications with cardioprotective and renoprotective effects.

Several limitations of our study deserve mention. First, we only had participant reported prescriptions and did not have information on prescription adherence which limits our ability to differentiate between true treatment-resistant hypertension and uncontrolled hypertension as the result of medication noncompliance [24]. Second, medications are assessed at a single timepoint due to the cross-sectional nature of NHANES. A time-updated analysis could account for changing patterns of comorbidities, medications, and BP over time and may find different associations. Third, we did not have information on medication doses. Physician prescription patterns, such as the use of low dose combination antihypertensive medications, may incorrectly assign participants to the aTRH category and bias the observed associations. Fourth, we only assessed cardiovascular mortality and did not have information on cardiovascular events. These limitations of our study are balanced by its strengths including its large sample size representative of the U.S. population, inclusion of racial/ethnic minorities, broad age range, rigorous data collection and extensive information on covariates, large number of events, and near-complete mortality follow-up using the NDI. The results of our study are generalizable to non-institutionalized U.S. adults.

## Conclusions

In conclusion, in the US population, aTRH is common with 7.6 million affected adults and is associated with higher risk of cardiovascular disease mortality. In particular, the risk of cardiovascular disease mortality remains high among those with controlled BP on 3 medications (non-aTRH) or ≥ 4 medications (controlled aTRH), groups not generally considered at high risk. Future risk reduction interventions should consider focusing on these high-risk groups.

## Additional file

Additional file 1: **Table S1.** Cumulative Incidence of All-Cause Mortality among NHANES participants with aTRH and controlled hypertension. (DOCX 16 kb)
