# Supplementary material for: Resistant hypertension and cardiovascular disease mortality in the US: results from the National Health and Nutrition Examination Survey (NHANES)
Source: BMC Nephrol. 2019 Apr 25;20:138. doi: 10.1186/s12882-019-1315-0 (PMC6485047; doi:10.1186/s12882-019-1315-0)
Supplement: Supplementary file 1 — Table S1. Cumulative Incidence of All-Cause Mortality among NHANES participants with aTRH and controlled hypertension. (DOCX 16 kb) [file 12882_2019_1315_MOESM1_ESM.docx]

Supplemental Table 1: Association of aTRH with All-Cause Mortality among NHANES (1988-1994 and 1999-2010) Participants

|  | Unweighted^1^ | | Weighted^2^ | | Crude IR^2^ (95% CI)  Per 1000 PY | Model 1: Unadjusted^3^ | | Model 2^4^ | | Model 3^5^ | | Model 4:  Fully Adjusted^6^ | |
| --- | --- | --- | --- | --- | --- | --- | --- | --- | --- | --- | --- | --- | --- |
|  | N (%) | Deaths (%) | N in millions (%) | Deaths in millions (%) |  | HR | p | HR | p | HR | p | HR | p |
| Non-aTRH | 4835 (76.1) | 1112 (23) | 29.04 (79.3) | 5.25 (18.1) | 24.3 (22.2, 26.5) | Reference |  | Reference |  | Reference |  | Reference |  |
|  |  |  |  |  |  |  |  |  |  |  |  |  |  |
| Any aTRH | 1522 (23.9) | 565 (37.1) | 7.6 (20.7) | 2.5 (33) | 50 (44.3, 56.5) | 2.12 (1.81, 2.49) | <0.001 | 1.48 (1.27, 1.72) | <0.001 | 1.33 (1.13, 1.57) | <0.001 | 1.15 (0.97, 1.36) | 0.115 |
| Controlled | 432 (6.8) | 122 (28.2) | 2.27 (6.2) | 0.57 (25) | 48.2 (38.2, 60.8) | 2.21 (1.7, 2.87) | <0.001 | 1.64 (1.27, 2.12) | <0.001 | 1.35 (1.01, 1.8) | 0.044 | 1.3 (0.98, 1.73) | 0.073 |
| Uncontrolled | 1090 (17.1) | 443 (40.6) | 5.33 (14.5) | 1.94 (36.4) | 50.5 (44.5, 57.4) | 2.1 (1.78, 2.48) | <0.001 | 1.44 (1.24, 1.67) | <0.001 | 1.33 (1.13, 1.56) | <0.001 | 1.11 (0.93, 1.33) | 0.235 |
|  |  |  |  |  |  | p-trend |  | p-trend |  | p-trend |  | p-trend |  |

Abbreviations: aTRH, apparent treatment-resistant hypertension; IR, incidence ratio

^1^ Unweighted refers to the actual number (%) of the NHANES participants from the final cohort.

^2^ Weighted refers to the projected numbers in the US populations and accounts for NHANES design and sampling weights.

HR from Cox proportional hazards models with adjustment as follows:

^3^Model 1 was unadjusted.

^4^Model 2 adjusted for age, sex and race.

^5^Model 3 adjusted for variables in Model 2 + body mass index (spline at 18 kg/m^2^ and 25 kg/m^2^), history of diabetes and cardiovascular disease, smoking status, serum total cholesterol, and ln-C-reactive protein (natural log transformed)

^6^Model 4 adjusted for variables in Model 3 + eGFR CKD EPI (spline at 60 and 90) and ln-ACR
